# Hierarchical Virtual Screening of Potential New Antibiotics from Polyoxygenated Dibenzofurans against *Staphylococcus aureus* Strains

**DOI:** 10.3390/ph16101430

**Published:** 2023-10-09

**Authors:** Lana P. S. Oliveira, Lúcio R. Lima, Luciane B. Silva, Jorddy N. Cruz, Ryan S. Ramos, Luciana S. Lima, Francy M. N. Cardoso, Aderaldo V. Silva, Dália P. Rodrigues, Gabriela S. Rodrigues, Aldo A. Proietti-Junior, Gabriela B. dos Santos, Joaquín M. Campos, Cleydson B. R. Santos

**Affiliations:** 1 Graduate Program in Biotechnology and Biodiversity-Network BIONORTE, Federal University of Amapá, Macapá 68903-419, Brazil; lanaoliveira2013@gmail.com (L.P.S.O.); ryanquimico@hotmail.com (R.S.R.); francy.cardoso@unifap.br (F.M.N.C.); aderaldosilva14@gmail.com (A.V.S.); aldo.proietti@unifap.br (A.A.P.-J.); 2 Laboratory of Modeling and Computational Chemistry, Department of Biological and Health Sciences, Federal University of Amapá, Macapá 68902-280, Brazil; luciorolima@gmail.com (L.R.L.); luciaanebarros@hotmail.com (L.B.S.); jorddynevescruz@gmail.com (J.N.C.); 3 Graduate Program in Network in Pharmaceutical Innovation, Federal University of Amapá, Macapá 68902-280, Brazil; 4 Graduate Program in Medicinal Chemistry and Molecular Modeling, Health Science Institute, Federal Univesity of Pará, Belém 66075-110, Brazil; 5 Special Laboratory of Applied Microbiology, Department of Biological and Health Sciences, Federal University of Amapá, Macapá 68902-280, Brazil; lucianasampaio@unifap.br; 6 Laboratory of Bacterial Enteric Pathogens, Oswaldo Cruz Foundation, FIOCRUZ, Rio de Janeiro 21045-900, Brazil; dalia.rodrigues@ioc.fiocruz.br; 7 Graduate Program in Health Sciences, Institute of Collective Health, Federal University of Western Pará, Santarém 68270-000, Brazil; gabrielastsrodrigues@gmail.com (G.S.R.); gabriela.bds@ufopa.edu.br (G.B.d.S.); 8 Department of Pharmaceutical and Organic Chemistry, Faculty of Pharmacy, Institute of Biosanitary Research ibs. GRANADA, University of Granada, 18071 Granada, Spain; jmcampos@ugr.es

**Keywords:** *Staphylococcus aureus*, polyoxygenated dibenzofurans, molecular docking, ADME/Tox properties

## Abstract

*Staphylococcus aureus* is a microorganism with high morbidity and mortality due to antibiotic-resistant strains, making the search for new therapeutic options urgent. In this context, computational drug design can facilitate the drug discovery process, optimizing time and resources. In this work, computational methods involving ligand- and structure-based virtual screening were employed to identify potential antibacterial agents against the *S. aureus* MRSA and VRSA strains. To achieve this goal, tetrahydroxybenzofuran, a promising antibacterial agent according to in vitro tests described in the literature, was adopted as the pivotal molecule and derivative molecules were considered to generate a pharmacophore model, which was used to perform virtual screening on the Pharmit platform. Through this result, twenty-four molecules were selected from the MolPort^®^ database. Using the Tanimoto Index on the BindingDB web server, it was possible to select eighteen molecules with greater structural similarity in relation to commercial antibiotics (methicillin and oxacillin). Predictions of toxicological and pharmacokinetic properties (ADME/Tox) using the eighteen most similar molecules, showed that only three exhibited desired properties (LB255, LB320 and LB415). In the molecular docking study, the promising molecules LB255, LB320 and LB415 showed significant values in both molecular targets. LB320 presented better binding affinity to MRSA (−8.18 kcal/mol) and VRSA (−8.01 kcal/mol) targets. Through PASS web server, the three molecules, specially LB320, showed potential for antibacterial activity. Synthetic accessibility (SA) analysis performed on AMBIT and SwissADME web servers showed that LB255 and LB415 can be considered difficult to synthesize and LB320 is considered easy. In conclusion, the results suggest that these ligands, particularly LB320, may bind strongly to the studied targets and may have appropriate ADME/Tox properties in experimental studies.

## 1. Introduction

Antimicrobial resistance is among the top 10 global health threats (Organization, n.d.). Resistance of bacteria to antibiotics is an urgent global public health and socioeconomic problem. Murray et al. [1] estimated that in 2019, 4.95 million deaths were associated with bacterial resistance to antibiotics. Among the various existing human pathogens, *Staphylococcus aureus* is one of the most interesting with regard to multidrug resistance, due to its intrinsic virulence with the ability to cause various infections. Mortality in cases of methicillin-resistant *S. aureus* bacteremia, for example, caused more than 100,000 deaths in 2019.

*S. aureus* is a Gram-positive bacterium responsible for skin and soft tissue infections [2]. Currently used naturally occurring antimicrobials (penicillins, cephalosporins, glycopeptides and β-lactams) with therapeutic potential have been shown to be increasingly ineffective in eradicating the more virulent strains of *S. aureus* resistant to methicillin (MRSA) [3].

Natural products are known as essential sources commonly used in drug discovery, providing a wide variety of pharmacologically active new structures and making it possible to obtain semisynthetic derivatives with different mechanisms of action [4]. Previous works showed that dichloromethane extracts from the South American species *Achyroclyne satureoides* (Asteraceae) presented remarkable antibacterial activity. The compound responsible for biological activity was identified as achyrofuran [5], a prenylated polyoxygenated dibenzofuran, which has shown antibacterial activity against a range of clinically relevant Gram-positive bacteria, including MRSA strains [6]. The compound extracted from the leaves of *Pilidiostigma glabrum* [7] and rhodomyrtoxin B originally from *Rhodomyrtus macrocarpa*, both dibenzofurans, also showed high antibacterial activity [8,9]. Nevertheless, according to Oramas-Royo et al. [10] there is still no conclusive information about the properties associated with the structure–activity relationships of dibenzofurans. For this reason, rational drug design strategies and tools using medicinal chemistry and molecular modeling are essential [11,12].

Taking into account the context described above, this work was planned and developed with the aim of identifying new chemical entities with antibacterial activity against MRSA strains, from symmetrical polyoxygenated dibenzofurans. The molecules were 2-methyl-1-(2,4,6-trihydroxyphenyl)-1-butanone and 2-methyl-1-(2,4,6-trihydroxy-3-propylphenyl) butanone, which showed biological activity for ATCC 25923 *S. aureus* (MRSA), followed by bioinformatics techniques (virtual screening, ADME/Tox predictions, biological activity, molecular docking and molecular dynamic simulations, synthetic accessibility, lipophilicity and water solubility predictions) [13,14].

## 2. Results and Discussion

### 2.1. Pharmacophoric Model Generation

**Figure 1:**
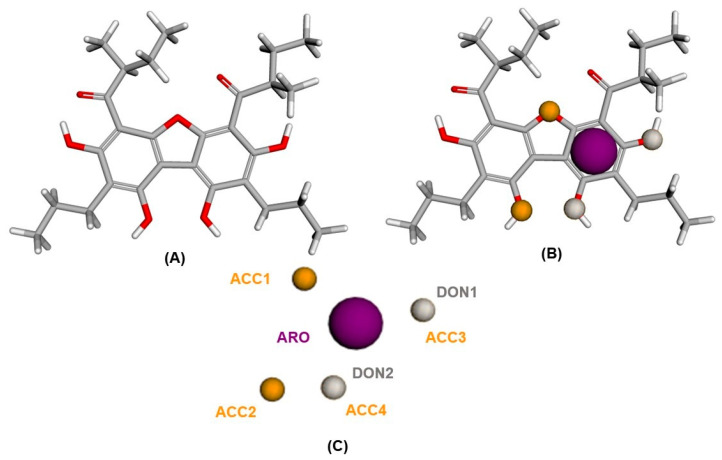
Pharmacophoric model: (**A**) Tetrahydroxybenzofuran molecule (pivot) 3D. (**B**) Pharmacophoric characteristics positioned in the tetrahydroxybenzofuran molecule (pivot). (**C**) Pharmacophoric characteristics: one aromatic (ARO), four hydrogen acceptors (ACC) and two hydrogen donors (DON). Oxygen atoms in red, carbon atoms in gray and hydrogen atoms in white.

The pharmacophoric model was obtained (Figure 1B) from the alignment of the tetrahydroxybenzofuran molecule (pivot) with the nine selected molecules (Figure 1A). The best alignment score was 32.476. Other in silico studies with similar method showed lower alignment values than our work [15,16], as well as works that looked for antimicrobial activity [17,18], indicating good quality of the pharmacophoric alignment.

This alignment gave rise to seven pharmacophoric characteristics: four hydrogen bond acceptors (ACC), two hydrogen bond donors (DON), one aromatic (ARO). Thus, it was confirmed that the aromatic pharmacophore ring (ARO) is located in the structure of benzofuran. The hydrogen bond donors (DON1 and DON2) are found on two hydroxyl groups attached to the aromatic ring of benzofuran. Three hydrogen bond acceptors (ACC2, ACC3 and ACC4) are also located on hydroxylated groups attached to the aromatic ring. Finally, one of the hydrogen bond acceptor pharmacophores (ACC1) is located on the oxygen atom of the furan ring of the structure. It is important to emphasize that the pharmacophoric characteristics of DON 1 and ACC 3 are in the same position in the molecule. Moreover, DON 2 and ACC 4 are also positioned in the same way (Figure 1C). The pharmacophore model obtained was submitted to obtain the spatial coordinates of the pharmacophore. The aligned molecules shared spatial characteristics, generating a model with the following coordinates (Table S1).

### 2.2. Pharmacophore-Based Virtual Screening

The range between the minimum and maximum values of each physicochemical property was used as a filter; furthermore, only the Top100 molecules were selected [19] (Table 1). The pharmacophoric model was satisfactory at this stage of the virtual screening process, as we obtained 100 previously selected molecules, with 76 molecules excluded due to their high RMSD values. Thus, only 24 molecules, with RMSD values ranging from 0.164 to 0.374, from this set were selected for the next analyzes.

### 2.3. Similarity of Tanimoto

At this stage, the 24 molecules selected in the previous analysis were compared according to the similarity and diversity of the set, with the tetrahydroxybenzofuran molecule (pivot) and with the commercial antibiotics methicillin and oxacillin.

Analyzing the Tanimoto indexes in comparison with the tetrahydroxybenzofuran molecule (pivot), it is noted that they ranged from 0.371 (MolPort-035-706-257) to 0.426 (Mol-Port-039-052-415); regarding methicillin, the values ranged from 0.225 (MolPort-035-706-258 and Mol-Port-035-706-259) to 0.275 (MolPort-039-338-750); and in relation to oxacillin from 0.199 (MolPort-039-052-415) to 0.276 (MolPort-005-945-312) (Table 2).

The molecules MolPort-039-052-415 and MolPort-001-741-320 showed greater similarity in relation to the tetrahydroxybenzofuran molecule (pivot). Analyzing the similarity with methicillin, the molecules Mol-Port-039-338-750 and MolPort-005-945-312 can be highlighted. Examining the obtained values in relation to oxacylin, the MolPort-005-945-312 and MolPort-039-338-750 molecules show greater similarity.

After this process, molecules with Tanimoto index values greater than or equal to 0.37 were selected, leaving only 18 molecules for carrying out pharmacokinetic and toxicological predictions (ADME/Tox).

### 2.4. Predictions of Toxicological and Pharmacokinetic Properties of the New Hits

The 18 molecules were subjected to predictions of toxicological properties. Thus, it was possible to predict carcinogenic activity of molecules in rats and mice. This prediction can be “positive”, meaning that there is no carcinogenicity, or “negative”, indicating the presence of this toxicological effect [20] (Table 3). It is observed that the molecule tetrahydroxybenzofuran (pivot) presented a negative result for mice and positive for rats. The two commercial compounds oxacillin and methicillin showed negative results for rats; however, oxacillin showed positive results for mice. Out of the molecules tested, only two (02) did not show carcinogenic properties, testing positive for mice and rats. The Ames test is a method that can determine, using bacteria, whether a molecule or chemical product has mutagenic capacity for the genetic material of the tested organism [17]. From the 18 molecules analyzed, nine (09) were considered mutagenic (Table 3).

Regarding the pharmacokinetics properties, the tetrahydroxybenzofuran molecule (pivot) showed a value above 90% for HIA (human intestinal absorption), the commercial compound methicillin showed values above 87%, while the commercial compound oxacillin had a percentage lower than 0.4%. This result corroborates the clinical observation that oxacilin does not present with oral administration. All other molecules show HIA greater than 84%, with the highest value being 92.40% (Table 4). The Caco-2 (human colorectal carcinoma) cell lines were used extensively to predict the molecules’ capability to cross the intestinal epithelium.

Once these cells have morphological and functional properties similar to human enterocytes, this model is a useful tool to predict the oral absorption of molecules in earlier stages of drug discovery [21]. The permeability of Caco-2 cells was investigated considering values above 500 nm·s^−1^ good and below 25 nm·s^−1^ bad [22]. All molecules, including the tetrahydroxybenzofuran molecule (pivot) and the commercial ones, showed values below 25 nm·s^−1^ for cellular permeability.

Plasma protein binding (PPB) is an important property that influences the pharmacokinetic and toxicokinetic of drugs. Normally, plasma proteins play a crucial role in drug distribution once they function as carriers of molecules from the site of absorption to the molecular target. However, extensive PPB affects drug clearance, metabolism, efficacy and safety [23].

Drug interactions can occur through a drug strongly bound to plasma proteins, as it can displace another drug and dramatically increase its free drug concentration (unbound portion), leading to adverse effects. In the literature, a percentage higher than 90% is considered high PPB; therefore, satisfactory results are found below this cutoff [20]. Both commercial drugs, oxacillin and methicillin, demonstrated weak PPB, with values lower than 90%. However, all other molecules, including the tetrahydroxybenzofuran molecule (pivot), showed extensive PPB values (see Table 5).

Predicting the blood–brain barrier permeation (C_brain_/C_blood_) is an important descriptor, since it estimates whether a compound is able to cross this compartment and act on the central nervous system (CNS). Molecules that do not exert a main pharmacological effect on the CNS must have blood–brain barrier permeation values less than 1 (C_brain_/C_blood_ < 1), as any value above is an indication that the compound is in high concentration both in the blood and in the brain, causing adverse effects [24,25]. Note that the commercial compounds and only three molecules fit the values described as acceptable in the literature; see Table 5.

### 2.5. Biological Activity Prediction

At this stage, only molecules with satisfactory results in the toxicological and pharmacokinetic analyses were submitted to the prediction of biological activity through the PASS online server. The quantitative parameter presents Pa as the probability of the compound being active and Pi as the probability of it being inactive; therefore, the activity was considered possible when Pa > Pi. Pa values close to 1 and Pi values close to 0 indicate a greater probability of molecules being active in experimental studies [26].

The commercial compounds methicillin and oxacillin were previously tested to assess the server’s degree of confidence; as expected, both showed antibacterial activity (Pa = 0.671 and Pa = 0.684, respectively) [27]. The tetrahydroxybenzofuran molecule (pivot) showed a Pa value equal to 0.465 for antibacterial activity on *S. aureus* strains, which corroborates with experimental works [10]. The MolPort-001-741-320 molecule showed potential for antibacterial activity, as it was observed that the value of Pa (0.487) is higher when compared to pivot; see Table 6.

The prediction of antibacterial activity via Antibac-Pred showed that the molecule tetrahydroxybenzofuran (pivot), oxacilin, methicillin and the molecule Mol-Port-001-741-320 showed activity against *S. aureus* (Pa ≥ 0.344). To facilitate reading, the nomenclature code of hits found in the MolPort database was standardized to LB420 (MolPort-001-741-320), LB255 (MolPort-035-706-255) and LB415 (MolPort-039-052-415), as shown in Table 6.

The parameter of probability of being active (Pa) reflects, first of all, the similarity of the molecule under prediction with the structures of the molecules, which are the most typical in a subset of “active” in the training set of Antibac-Pred. Therefore, there is no direct correlation between the Pa values and the quantitative characteristics of the activities [28,29]. Even active and potent compounds, whose structure is not typical of the “active” structures of the training set, can obtain a low Pa value and even Pa < Pi during prediction.

Analyzing the results of toxicological, pharmacokinetic and biological activity predictions, only molecules LB420 (MolPort-001-741-320), LB415 (MolPort-039-052-415 and LB255 (Mol-Port-035-706-255) showed satisfactory results to follow for further analysis. After selecting promising molecules, this study proceeded to structure-based virtual screening through molecular docking simulations.

### 2.6. Molecular Binding Mode

The biding sites were determined based on the crystallographic pose data of the complexed ligands (QZN and 0Y5) with the specific molecular targets from *S. aureus*, the penicillin-binding protein—PBP2a (PDB: 4CJN) and thymidylate kinase—TMK (PDB:4GSY), respectively. Validation occurred through the evaluation of the root mean square deviation (RMSD) between the pose of the crystallographic ligand (complex obtained using X-ray crystallography) with the theoretical ligand (computational); thus, the approach was used to select the active site based on biological studies.

**Figure 2:**
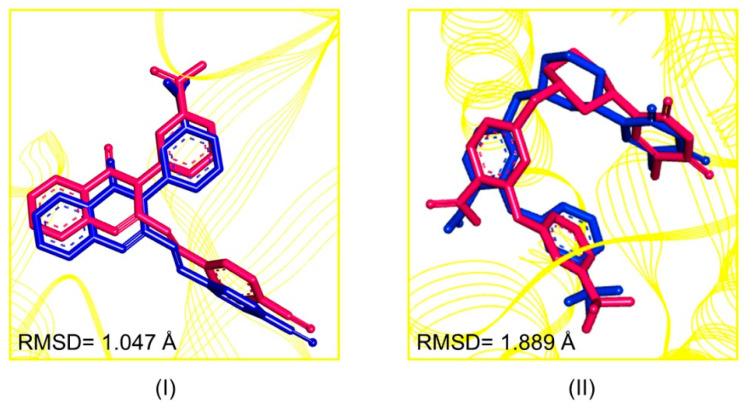
Overlapping of crystallographic ligands (pink) and those obtained using redocking (blue): QZN (**I**) and 0Y5 (**II**).

The RMSD results were 1.047 Å for the QZN ligand and 1.889 Å for the 0Y5 ligand, as can be seen in Figure 2. When the RMSD value obtained is equal to or less than 2.0 Å, it is considered a satisfactory result and the methodology of molecular docking is validated [28].

High-level resistance to β-lactam antibiotics in methicillin-resistant Staphylococcus aureus (MRSA) is due to expression of penicillin-binding protein 2a (PBP2a), a transpeptidase that catalyzes cell wall crosslinking in the face of the challenge from β-lactam antibiotics. The activity of this protein is regulated by allostery at a site 60 Å from the active site, where crosslinking of cell wall takes place [30].

Bouley et al. [31] determined the crystallographic structure of the complex of the quinazolinone ligand (QZN) with the penicillin-binding protein (PBP2a), obtaining a high resolution of 1.95 Å. Quinazolinone is located at the allosteric site of PBP2a. The critical interaction of ligands such as peptidoglycan, derived from the cell wall, at the allosteric site causes the opening of the active site, which allows for catalysis by PBP2a. The allosteric domain includes residues Ser27-Pro326, where the N-terminal domain (Ser27-Asn138) and the allosteric domain (Thr139-Pro326) are found. The main interactions of quinazolinone at the allosteric site are the following: salt bridge with Val273 and Asn316, Pi stacking interactions with Val105 and Asp297. It has been shown that PBP2a has two binding sites, an allosteric site and an active site separated by 60 Å. The binding of an allosteric effector can influence protein function and predisposes PBP2a to inactivation; therefore, allosteric binding sites can be targets for new drugs [32].

TMK is a nucleotide kinase that catalyzes the phosphorylation of deoxythymidine monophosphate (dTMP) to deoxythymidine diphosphate (dTDP) using ATP as a co-substrate. This is a necessary step in the biosynthesis of deoxythymidine triphosphate (dTTP) for DNA synthesis. This makes TMK an essential enzyme and a very attractive target for therapeutic intervention [33].

Martínez-Botella et al. [33], in the search for selective and potent inhibitors of TMK, synthetized new analogs from piperidinylthymine, and determined the crystallographic structure of an inhibitor (0Y5) co-crystalized to *S. aureus* TMK. When anchored in the active site, the 0Y5 ligand performs multiple hydrogen bonding interactions with residues Agr70, Ser97 and Gln101, in addition to Pi stacking interactions with Phe66.

**Figure 3:**
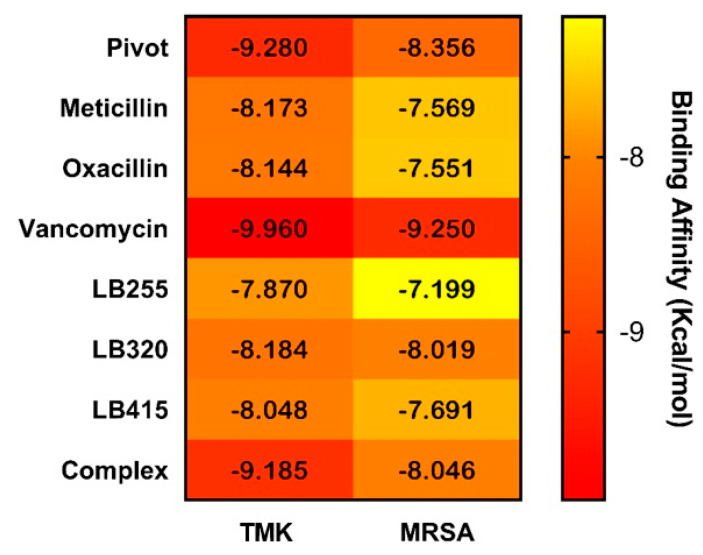
Heatmap plot of binding affinity (ΔG) values of pivot (tetrahydroxybenzofuran) and promising molecules (LB255, LB320 and LB415) compared to commercial compounds (methicillin, oxacillin and vancomycin) and co-crystallized ligand at the active sites of TMK and PBP2a-MRSA targets.

In order to assess whether the three promising molecules from the pharmacophore-based virtual screening had a higher binding affinity than the complexed ligands (QZN and 0Y5), the commercial compounds (methicillin, oxacillin and vancomycin) and the tetrahydroxybenzofuran molecule (pivot) of the study, both for penicillin-binding protein (PBP2a) and for the thymidylate kinase enzyme (TMK). Taking into account the heatmap graph presented in Figure 3, the promising molecule LB320 was the one that presented the best binding affinity results when compared to the control compounds, complexed compounds and the pivot molecule, followed by the molecules LB415 and LB255.

It is noted that the promising molecule LB320 showed higher binding affinity results than methicillin and oxacillin controls on TMK and PBP2a-MRSA targets. In the PBP2a-MRSA target, the promising molecule LB320 showed very close binding affinity to the tetrahydroxybenzofuran molecule (pivot) of this study, showing a difference of ±0.337 Kcal/mol. The high binding affinity of vancomycin, superior to the complexed ligands, was also observed. The other promising molecules (LB255 and LB415) in the study showed binding affinity values lower than −7 Kcal/mol; nevertheless, further investigation must be carried out to find out whether these ligands can bind to the PBP2a-MRSA active site [34].

**Figure 4:**
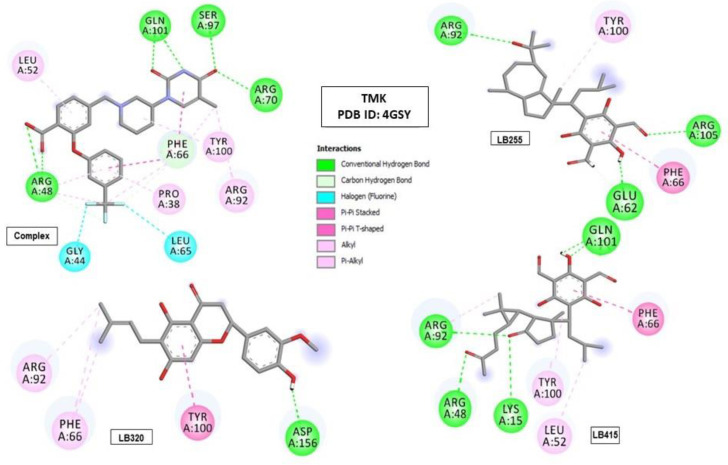
Interactions of the co-crystallized ligand (complex) and the tested molecules (LB255, LB320 and LB415) with the amino acid residues of the Gram-positive bacterial enzyme thymidylate kinase (TMK) (PDB ID 4GSY).

In the active site of the TMK enzyme, the promising molecules (LB255, LB320 and LB415) showed some interactions with amino acid residues that corroborate the interactions of the crystallographic ligand. The compound LB255 showed Pi–alkyl hydrophobic interactions with the Tyr100 residue and Pi–Pi T-shaped with the Phe66 residue. The compound LB320 showed Pi–alkyl hydrophobic interactions with residues Arg92 and Phe66. On the other hand, the compound LB415 kept the hydrogen bonds with Arg48 and Gln101, also performed Pi–alkyl hydrophobic interactions with residues Leu52 and Tyr100, as well Pi–Pi T-Shaped hydrophobic interactions with the residue Phe66, presenting a similar biding mode to 0Y5, the co-crystallized ligand (Figure 4 and Table 7).

In recent decades, the discovery of new antibiotics has been a challenge for science, industry and academia, as the need to treat infections of Gram-positive bacteria resistant to current drugs is urgent [33]. The bacterial enzyme thymidylate kinase (TMK) is found at the junction of the de novo and rescue pathways of thymine triphosphate (dTTP) synthesis. In view of its low sequence identity (22%) with the human enzyme, this enzyme becomes a very attractive therapeutic molecular target for selective inhibition of microorganism DNA synthesis [34].

**Figure 5:**
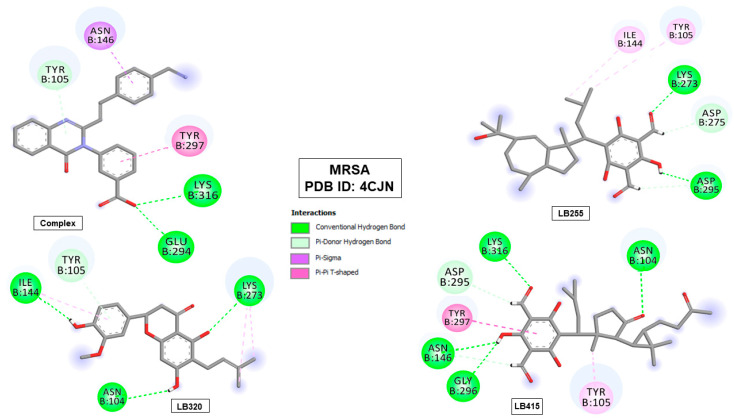
Interactions of the co-crystallized ligand (complex) and the tested molecules (LB255, LB320 and LB415) with penicillin-binding protein (PBP2a-MRSA) amino acid residues (PDB ID 4CJN).

In the allosteric site of penicillin-binding protein (PBP2a-MRSA), the promising compounds also performed some interactions with amino acid residues that corroborate the interactions of the co-crystallized ligand. The LB320 molecule showed a carbon–hydrogen-type interaction with Tyr105 residue. The LB415 molecule performed a Pi–Pi T-shaped hydrophobic interaction with the Tyr297 residue and conventional hydrogen bond with the Lys316 residue (see Figure 5 and Table 8).

Penicillin, methicillin and β-lactam antibiotics are analogous structures of penicillin-binding proteins (PBPs) whose role is to catalyze the formation of peptide crosslinks (transpeptidation) between glycan chains of the cell wall. The covalent inhibition of PBPs leads to the weakening of the cell wall and eventually its death [35].

**Figure 6:**
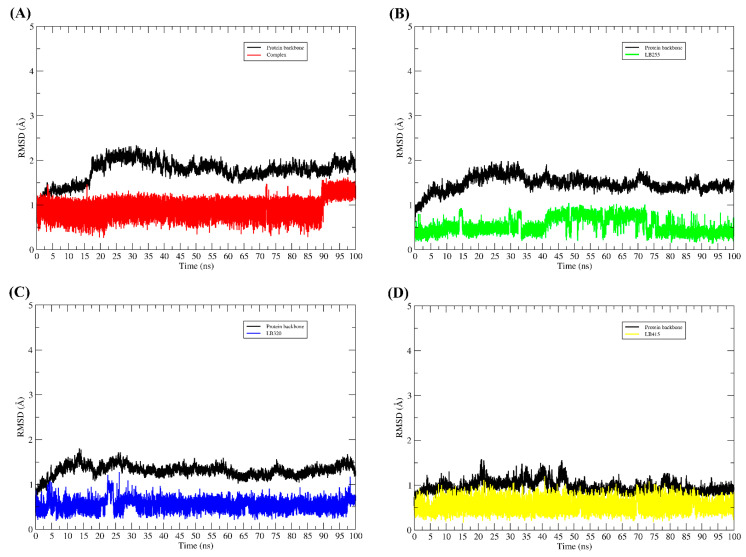
RMSD plot along the path of molecular dynamics simulations. RMSD graphs for 100 ns of MD simulations. In all the figures, the RMSD plot of the TMK backbone is represented by the color black, while the RMSD of the ligands is represented in different colors. (**A**) RMSDs of complex (co-crystallized ligand) system, (**B**) RMSDs of LB255 system, (**C**) RMSDs of LB320 system, (**D**) RMSDs of LB415 system.

**Figure 7:**
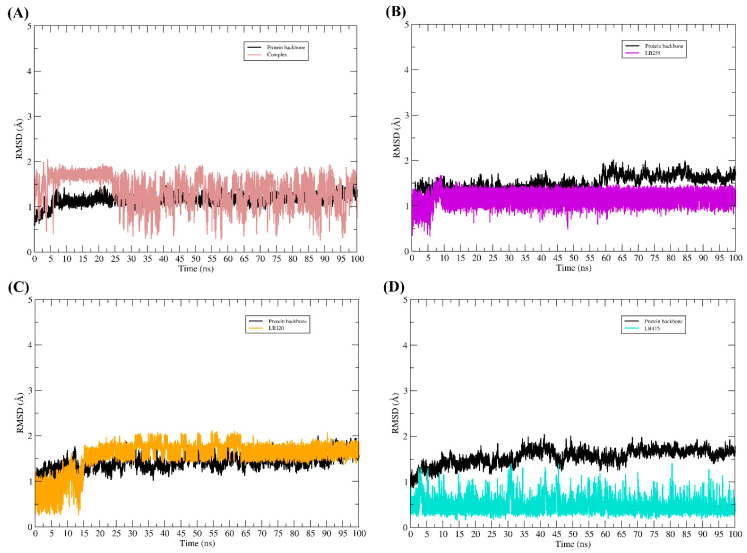
RMSD plot along the path of molecular dynamics simulations. RMSD graphs for 100 ns of MD simulations. In all the figures, the RMSD plot of the PBP2a backbone is represented by the color black, while the RMSD of the ligands is represented in different colors. (**A**) RMSDs of complex system (co-crystallized ligand), (**B**) RMSDs of LB255 system, (**C**) RMSDs of LB320 system, (**D**) RMSDs of LB415 system.

The complexes obtained using molecular docking served as a starting point for molecular dynamics simulations. For each TMK and PBP2a complex interacting with the ligands, 100 ns of md simulations were generated. The RMSD graphs for each complex can be seen in Figure 6 and Figure 7.

Throughout the molecular dynamic simulations, all ligands remained interacting with the binding pocket. None of them detached from the target protein and, according to the profile of the RMSD graphs, their conformational changes in the binding cavity were not sudden; thus, we can infer that the results obtained using molecular docking were satisfactory, since the mode of interaction obtained in docking did not undergo sudden changes, therefore, without major change in the binding mode of the compounds.

After the dynamic simulations, the complexes were again evaluated for their affinity energy. All complexes demonstrated that they can spontaneously remain interacting, as the affinity energy values obtained with the MM/GBSA method were all negative, as we can see in Table 9.

### 2.7. Prediction of Synthetic Accessibility (SA)

AMBIT and SwissADME were used to evaluate the SA of LB255, LB320 and LB415 (Table 10). Only the LB320 molecule presented the predicted synthetic accessibility as easy, obtaining a score of 65.08%. LB255 and LB415 had SA reaching a score above 36.58% and 38.88%, respectively, indicating median accessibility for synthesis. In comparison, the accessibility prediction for pivot was 51.30%.

The result obtained using SwissADME for the LB320 showed an SA score of 30.88%. LB255 and LB415 presented, respectively, a score of 50.17% and 50.06 (see Table 10). Compared to pivot, which presented a SA score of 40.98%, the SA values were close, ranging between ±14.73 and ±12.43 for LB255 and LB415, respectively. LB255 and LB415 can be considered difficult to synthesize, considering the results obtained and the data found in the literature. Regarding the pivot, the LB320 molecule showed a variation of ±13.77 for AMBIT web server and ±10.11 for SwissADME.

### 2.8. Prediction of Lipophilicity and Water Solubility and Structure–Activity Relationship (SAR) of the Promising Molecule

The commercial compounds (methicillin and oxacillin) presented consensus LogP values below 2, which shows that they are water-soluble molecules, which helps in blood distribution since these molecules are administered intramuscularly/intravenously [36].

On the other hand, promising molecules showed particular characteristics in relation to LogP. The LB255 molecule was the closest to the commercial compounds, showing its consensual LogP lower than 2, which characterizes it as a water-soluble molecule. This is due to the fact that this molecule has few unsaturations and no aromatic groups in its structure. On the contrary, molecules LB320 and LB415 were a little more lipophilic (LogP > 2) when compared to the other compounds studied, therefore less soluble in water, which is confirmed by a greater number of unsaturations (carbonyl groups) in their structures (Table 11 and Table 12).

The three compounds with the most promising results at the end of the virtual screening were searched on SciFinder^®^ (https://scifinder.cas.org/ (accessed on 26 March 2023)). No additional information was found on the selected LB415 compound in the search; only information about some physical and chemical properties has already been reported in the MolPort database.

The LB255 molecule revealed important properties for Gram-positive bacteria such as Bacillus subtilis and Staphylococcus aureus for MRSA FAD209P strains [34]; primarily, the two carbonyl groups are essential for antibacterial activity [37].

The LB320 molecule having the presence of the methoxy group attached to the aromatic ring of the structure significantly alters the mechanism of action [38]. In addition, it revealed important properties against methicillin-resistant Staphylococcus aureus (MRSA) for similar structures for strains of MRSA 1903, MRSA 63718, MRSA 62097, MRSA 62059, MRSA 67755 and MRSA 1679, and promising synergistic activities with antibiotics [39]. Overall, the results of the present study suggest that selected compounds can be tested for biological activities with good evidence to reproduce the in silico results. Therefore, future studies are needed to confirm the antibacterial activity for Staphylococcus aureus strains of MRSA 700699 by these molecules.

## 3. Materials and Methods

**Figure 8:**
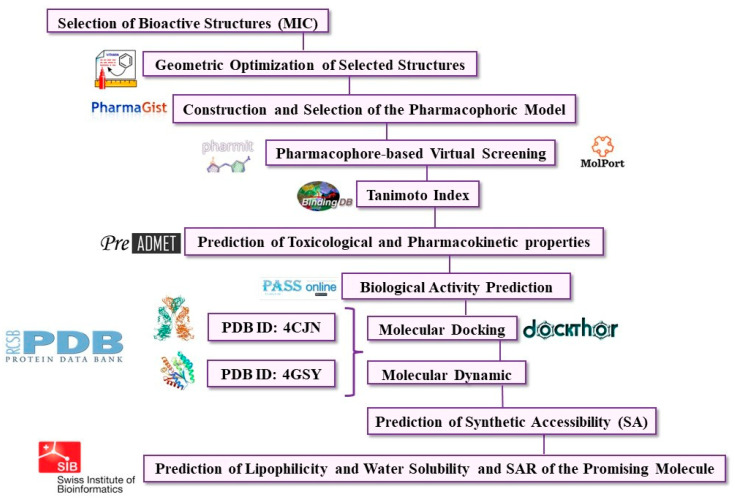
Flowchart of the methodological steps taken in this study.

All methodological steps and computational tools used in this study are summarized in Figure 8.

### 3.1. Selection of Molecules

**Figure 9:**
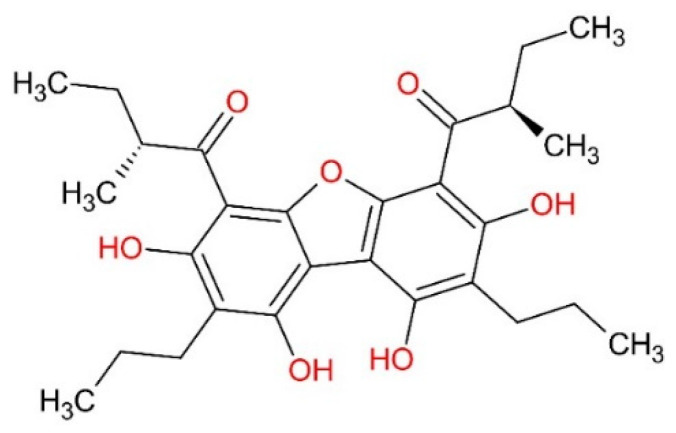
Two-dimensional structure of the pivotal tetrahydroxybenzofuran molecule.

**Figure 10:**
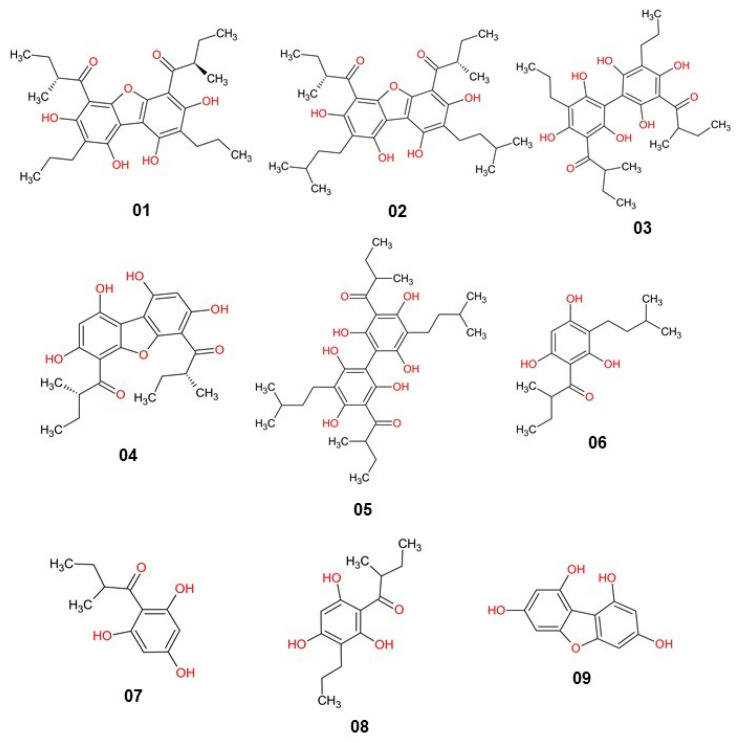
Molecular structures selected in this study, based on symmetrical polyoxygenated dibenzofurans, 2-methyl-1-(2,4,6-trihydroxyphenyl1)butanone and 2-methyl-1-(2,4,6-trihydroxy-3)-propylphenyl) butanone.

The molecules were selected based on the Minimum Inhibitory Concentration (MIC) values for ATCC 25923 S. aureus (MSSA—methicillin-susceptible S. aureus), ranging from 0.24 µg/mL to >30 µg/mL from the study by Oramas-Royo et al. [40] (Table 13). The molecules were arranged in ascending order of MIC (µg/mL), from the most active to the least active, respectively. The tetrahydroxybenzofuran (01) molecule was selected as the pivotal structure of this study, based on the lowest MIC value (Figure 9). Molecular structures were drawn using ChemSketch software. Molecular structures can be seen in Figure 10.

### 3.2. Geometric Optimization of Selected Structures

The optimization of the three-dimensional structure of the compounds was performed in ChemSketch software using molecular mechanics methods with the CHA-ARMM force field [15,16].

### 3.3. Construction of the Pharmacophoric Model

The PharmaGist web server (http://bioinfo3d.cs.tau.ac.il/pharma/php.php, accessed on 13 April 2021) was used to build several pharmacophoric models, where the best result was filtered from the obtained score values [41].

### 3.4. Pharmacophore-Based Virtual Screening

Pharmit has large prebuilt libraries generated from MolPort, ChEMBL, ChemDiv, PubChem and NCI Open Chemical Repository. Only the MolPort database presents more than 6.5 million compounds. In an attempt to reduce the number of molecules, Pharmit allows the user to add nonstructural filters constituted by molecular properties, such as molecular weight (MW), number of rotatable bonds (NRB), polar surface area (PSA), coefficient of lipophilicity (LogP), number of hydrogen bond donors and acceptors (HBD and HBA, respectively), as well as amount of aromatic groups. In this way, molecules can be filtered by specifying desired ranges for those molecular properties that are often used to recognize drug-like molecules [19].

In this step, the Pharmit online platform (http://pharmit.csb.pitt.edu/search.html, accessed on 15 April 2021) was used to search new hits based on selected pharmacophoric model. This web server is an interactive environment for virtual screening of large databases of compounds using pharmacophores, molecular shape and minimization of energy [42]. Thus, according the pharmacophoric characteristics, molecular parameter filters were applied, such as molecular weight (MW), number of rotatable bonds (RotB), lipophilicity (LogP), polar surface area (PSA), hydrogen bond acceptors (HBA), hydrogen bond donors (HBD), extracted from online platforms Molinspiration (https://www.molinspiration.com/, accessed on 30 September 2023) and ProTox-II (http://tox.charite.de/protox_II/, accessed on 15 April 2021). The Top100 structures of the MolPort^®^ company database (~7.9 million compounds) (https://www.molport.com/shop/index, accessed 20 April 2021) were obtained based on the filter of maximum and minimum values of the molecular descriptors, according to Dos Santos et al. [43].

### 3.5. Similarity of Tanimoto coefficient

The Tanimoto coefficient (Equation (1)) is a measure of similarity with values range from 0 to 1. This index represents the similarity between two compounds based on the bits (molecular fragments) of fingerprint, that is, the higher the value, the greater the similarity [44].

(1) $$Tanimoto\;coefficient=\frac{C}{\left(A+B-C\right)}$$

where *A* corresponds to the number of bits in *A*, while *B* corresponds to the number of bits in compound *B* and *C* to the number of common bits in compounds *A* and *B* [44]. The similarity calculation was performed on BindingDB (http://www.bindingdb.org/bind/index.jsp, accessed on 20 April 2021), a web-accessible database. At this stage, tetrahydroxybenzofuran (pivot) and two approved drugs, methicillin and oxacillin, served as reference ligands [45]. Thus, the hits provided by pharmacophore-based virtual screening were compared to reference ligands, according to studies carried out by Ferreira et al. [22]. After this step, the hits most similar to the reference compounds were submitted to toxicological and pharmacokinetic predictions.

### 3.6. Prediction of Pharmacokinetic and Toxicological Properties of the New Hits

The PreADMET web server (https://preadmet.webservice.bmdrc.org/adme/, accessed on 20 April 2021) was used to evaluate the pharmacokinetic properties of the new hits using oxacillin and methicillin as controls. Thus, the following properties were evaluated: human intestinal absorption (HIA), in vitro Caco-2 permeability (P_Caco-2_), plasma protein binding (PPB) and blood–brain barrier permeation (C_Brain_/C_Blood_). Toxicological properties such as carcinogenicity and the Ames test were also calculated on this server. The ProTox-II web server (https://tox-new.charite.de/protox_II/index.php?site=compound_search_similarity, accessed on 23 April 2021) was used to evaluate the median lethal dose (LD50) of compounds provided using pharmacophore-based virtual screening [46].

### 3.7. Biological Activity Prediction of the New Hits

The prediction of the biological activity of the structures was performed using the PASS server (http://www.akosgmbh.de/pass/index.html, accessed on 17 September 2022), which predicts up to 2000 biological activities for chemical compounds with high accuracy (70–80%). This server provides two probabilities with values ranging between 0.000 and 1.000, Pa (probability of being active) and Pi (probability of being inactive) for each investigated target [26].

Prediction of antibacterial activity was obtained via Antibac-Pred (http://www.way2drug.com/antibac/, accessed on 17 September 2022). This web server allows the user to predict if a chemical compound is able to inhibit the growth of one or more than 353 species of bacteria at concentrations below 10,000 nM. The score for each compound is expressed as confidence in its activity, which is a difference between the probabilities that a chemical compound inhibits or does not inhibit the growth of a given bacteria. As confidence increases, the chances of the prediction being true are greater. Only activities with Pa > Pi (i.e., confidence > 0) are considered possible for a given compound [29].

### 3.8. Molecular Docking

The structures for the molecular docking study were prepared with the help of BIOVIA Discovery Studio^®^ v. 20.1.0 software [47] to remove residual water and cofactors. In this study, the molecular targets of the *S. aureus* organism, penicillin-binding protein 2 and thymidylate kinase enzyme were obtained from the Protein Data Bank (https://www.rcsb.org/, accessed on 20 September 2022), with the respective PDB ID codes: 4CJN (resolution 1.95 Å) [28] and 4GSY (resolution 1.71 Å) [33].

The validation of the molecular docking methodology occurred through the study of molecular redocking, where the crystallographic ligands themselves were submitted to the docking process using the DockThor web server (https://dockthor.lncc.br/v2/, accessed on 22 September 2022) [48] and calculated the RMSD (root mean square deviation) values of the crystallographic pose of the ligands in comparison to the computational study [49,50,51].

The x, y, z coordinates of the active site of each target were selected according to Table 14. The molecular docking simulation followed the preestablished protocol on the webserver [52,53] using the precision of the search algorithm in “Standard” mode, where 1,000,000 evaluations and 24 races are held. The analysis took into account the binding affinity results (Kcal/mol) and the molecular interactions of the receptors with the commercial compounds (vancomycin, methicillin and oxacillin) comparing the promising compounds and the complexed ligands of each target.

### 3.9. Molecular Dynamic Simulations

The RESP charges of the ligands were obtained with HF/6-31G [54], the parameters were created using Antechamber [55], being described by the GAFF [56]. Molecular dynamic (MD) simulations were performed using Amber 18 software [57,58]. The tLEaP module was used to add the missing hydrogens to protein structures. The PDB2PQR server (https://server.poissonboltzmann.org/pdb2pqr, accessed on 26 September 2022) [59] was used to determine the protonation state of protein residues. MD simulations were run using the 14SB force field [60]. TIP3P water molecules [61] were used to solvate the systems in an octahedron periodic box. The distance for the shear radius was 12 Å for all directions. Counterions were added to neutralize the system charges.

The sander.MPI module was used to perform energy minimizations. First, the water molecules and ions were optimized using 2000 cycles of the steepest descent and 3000 cycles of conjugate gradient. The position of receptor–ligand hydrogen atoms was then optimized using 4000 steps of steepest descent algorithm and 3000 steps of conjugate gradient. At the third stage, hydrogen atoms, water molecules, and ions were further optimized using 2500 steps of steepest descent algorithm and 3500 steps of conjugate gradient. All atoms were minimized using 3000 steps of the steepest descent algorithm and 3 steps of the conjugate gradient.

Then, the complexes were heated up to 300k in five steps for a total time of 500 ps. In the initial four steps, a harmonic force constant of 25 kcal/mol.Å^−2^ was used to constrain the heavy atoms. In the last step, the harmonic force constant was reduced to zero. To balance the systems, MD simulations of 5 ns were performed at 300 K without any restrictions. Finally, MD production simulations were run for a total time of 100 ns.

The particle mesh Ewald method [62] was used for the calculation of the electrostatic interactions and the bonds involving hydrogen atoms were restricted with the SHAKE algorithm [63]. The temperature control was performed with the Langevin thermostat [64] within a collision frequency of 2 ps^−1^.

### 3.10. Free Energy Calculations

The molecular mechanics/generalized Born surface area (MM/GBSA) method [51,65] was applied to estimate the receptor–ligand affinity energy. For our calculations, we used 500 snapshots of the last 5 ns of MD simulation.

The free energy was estimated according to Equation (2):

ΔG_bind_ = ΔE_MM_ + ΔG_solv_ − TΔS (2)

ΔG_bind_ is the affinity energy resulting from the sum of the total energy in the gas phase (ΔE_MM_), free energy of solvation (ΔG_solv_) and entropy (TΔS).

ΔE_MM_ is the sum of ΔE_internal_ (connections, angles and dihedra), ΔE_electrostatic_ (electrostatic contributions) and ΔE_vdW_ (van der Waals contributions), according to Equation (3):

ΔE_MM_ = ΔE_internal_ + ΔE_electrostatic_ + ΔE_vdw_ (3)

ΔG_solv_ can be obtained from the resolution of Equation (4):

ΔG_solv_ = ΔG_GB_ + ΔG_SASA_ (4)

where the polar contribution (ΔG_GB_) is calculated using the GB model and the nonpolar contributions (ΔG_SASA_) are determined from the calculation of the solvent-accessible surface area (SASA).

### 3.11. Synthetic Accessibility Prediction

Synthetic accessibility (SA) prediction of LB255, LB320, LB415 and pivot) was performed using AMBIT and SwissADME (http://www.swissadme.ch/, accessed on 1 October 2022). AMBIT uses the model for SA that represent different structural and topological features combined in an additive scheme. SA is issued with a score ranging from 0 to 100, where 100 is the molecule that is most easily synthesized [66]. The SwissADME runs on a score based on piecemeal analysis of the structures with the hypothesis that the more frequent a molecular fragment, the easier it is to obtain the molecule. The SA score range is set between 10 (easy synthesis) and 100 (very difficult synthesis) [67].

### 3.12. Prediction of Lipophilicity and Water Solubility and SAR of the Promising Molecules

Lipophilicity and water solubility values were predicted on the SwissADME webserver [68] This server has a large database where it is possible to accurately estimate physicochemical properties, lipophilicity, water solubility, pharmacokinetics, “drug-likeness” and medicinal chemistry properties. The analyses followed the methodological proposal of Sepay et al. [69] which consists of using the various predictive methods of LogP and LogS that the SwissADME makes available, such as: iLOGP, XLOP3, WLOGP, MLOGP, ESOL, the Ali method and the SILICOS-IT method (http://silicos-it.be.s3-website-eu-west-1.amazonaws.com/index.html, accessed on 1 October 2022). In this way, a consensual analysis of all informed descriptors is carried out. Therefore, the more diverse the prediction methods are, the more accurate the consensus value [70].

## 4. Conclusions

In this work, computational strategies of virtual screening based on pharmacophore and ligand were applied with the objective of identifying new chemical entities with antibacterial activity against *Staphylococcus aureus* MRSA strain, from symmetrical polyoxygenated dibenzofurans, 2-methyl-1-(2,4,6-trihydroxyphenyl-1) butanone and 2-methyl-1-(2,4,6-trihydroxy-3-propylphenyl)butanone. The main results obtained from the virtual screening, ADME/Tox, DM and affinity energy (MM/GBSA) were satisfactory, bearing in mind that these results were fundamental in the selection of the potential molecule. Analyses of lipophilicity and water solubility were essential to predict the hydrophilic and lipophilic character of the molecule. The molecular docking study was necessary for the selection of the three promising molecules based on the binding affinity values and the interactions with the amino acid residues present in the molecular targets used in this study.

## Figures and Tables

**Table 1:** Physical–chemical properties of selected compounds in maximum and minimum filters.

Chemical Structure	MW ^1^	RotB ^2^	PSA ^3^	Log*P* ^(a)^	Donor (HBD)	Acceptor (HBA)	Aromatic ^(b)^
01	484.58	10	128.20	7.12	4		3
						7	
02	540.69	12	128.20	8.51	4		3
						7	
03	502.60	11	155.50	6.75	6		2
						8	
04	400.42	6	128.20	5.07			3
					4	7	
05	558.71	13	155.50	8.26	6		2
						8	
06	280.36	6	77.70	4.71	3		1
						4	
07	210.23	3	77.70	2.44	3		1
						4	
08	252.31	5	77.70	3.93	3		1
						4	
09	232.19	0	94.00	1.98	4		3
						5	
Minimum	210.23	0	77.70	1.98	3	4	1
Maximum	558.71	13	155.50	8.51	6	8	3

^1^ MW = molecular weight; ^2^ RotB = rotatable bonds; ^3^ PSA = polar surface area; ^(a)^ Log*P* = lipophilicity; HBA = hydrogen bond acceptors; HBD = hydrogen bond donors; ^(b)^ PharmaGist.

**Table 2:** Tanimoto index of the selected compounds in relation to pivot and the commercials methicillin and oxacillin.

Chemical Structure	Tanimoto Index		
	Tetrahydroxybenzofuran (Pivot)	Methicillin	Oxacillin
MolPort-039-052-415	0.426	0.228	0.199
MolPort-001-741-320	0.421	0.262	0.250
MolPort-039-339-001	0.416	0.254	0.251
MolPort-001-742-504	0.416	0.253	0.249
MolPort-039-338-719	0.409	0.252	0.251
MolPort-039-338-651	0.409	0.251	0.250
MolPort-039-052-414	0.403	0.226	0.200
MolPort-039-338-750	0.401	0.275	0.269
MolPort-039-339-000	0.399	0.254	0.256
MolPort-005-945-435	0.397	0.251	0.238
MolPort-039-052-600	0.397	0.227	0.204
MolPort-035-706-258	0.394	0.225	0.205
MolPort-035-706-259	0.394	0.225	0.205
MolPort-028-610-187	0.393	0.250	0.240
MolPort-028-610-188	0.392	0.252	0.242
MolPort-005-945-312	0.388	0.266	0.276
MolPort-035-706-255	0.376	0.255	0.240
MolPort-035-706-257	0.371	0.234	0.225

**Table 3:** Predicted toxicological, carcinogenic and mutagenic properties of the structures.

Chemical Structure	Carcinogenicity ^(a)^		Ames Test ^(a)^	LD_50_^(b)^_(mg/kg)_	Class ^(b)^
	Rat	Mouse	Mutagenicity		
Tetrahydroxybutanefuran (pivot)	Negative	Positive	Mutagenic	1000	4
Oxacillin	Positive	Negative	Not mutagenic	5000	5
Methicillin	Negative	Negative	Not mutagenic	2880	5
MolPort-001-741-320	Negative	Positive	Mutagenic	2000	4
MolPort-001-742-504	Negative	Positive	Mutagenic	2000	4
MolPort-005-945-312	Negative	Negative	Not mutagenic	2000	4
MolPort-005-945-435	Negative	Negative	Not mutagenic	10	2
MolPort-028-610-187	Negative	Negative	Mutagenic	10	2
MolPort-028-610-188	Negative	Negative	Not mutagenic	10	2
MolPort-035-706-255	Negative	Positive	Not mutagenic	690	4
MolPort-035-706-257	Positive	Positive	Not mutagenic	690	4
MolPort-035-706-258	Negative	Positive	Mutagenic	400	4
MolPort-035-706-259	Negative	Positive	Mutagenic	400	4
MolPort-039-052-414	Negative	Positive	Mutagenic	1060	4
MolPort-039-052-415	Positive	Negative	Not mutagenic	1060	4
MolPort-039-052-600	Negative	Positive	Not mutagenic	400	4
MolPort-039-338-651	Negative	Positive	Mutagenic	2000	4
MolPort-039-338-719	Negative	Positive	Mutagenic	2000	4
MolPort-039-338-750	Positive	Positive	Not mutagenic	2000	4
MolPort-039-339-000	Positive	Negative	Mutagenic	2000	4
MolPort-039-339-001	Negative	Positive	Not mutagenic	2000	4

^(a)^ PreADMET; ^(b)^ ProTox-II; LD_50_ = median lethal dose. ^(b)^ Class 1: fatal if swallowed (LD_50_ ≤ 5); class 2: fatal if swallowed (5 < LD_50_ ≤ 50); class 3: toxic if ingested (50 < LD_50_ ≤ 300); class 4: dangerous if ingested (300 < LD_50_ ≤ 2000); class 5: may be harmful if swallowed (2000 < LD_50_ ≤ 5000); class 6: nontoxic (LD_50_ > 5000).

**Table 4:** Predicted absorption properties of the structures.

Chemical Structure	HIA% ^(a)^	P_Caco-2_ (nm/s) ^(b)^
Tetrahydroxybutanefuran (pivot)	90.04	20.23
Oxacillin	0.42	20.15
Methicillin	87.32	14.99
MolPort-001-741-320	90.45	7.82
MolPort-001-742-504	90.29	10.95
MolPort-005-945-312	88.58	12.02
MolPort-005-945-435	88.40	13.14
MolPort-028-610-187	92.38	17.18
MolPort-028-610-188	92.39	14.70
MolPort-035-706-255	86.88	18.19
MolPort-035-706-257	86.88	17.80
MolPort-035-706-258	86.88	17.80
MolPort-035-706-259	86.88	17.80
MolPort-039-052-414	91.59	20.06
MolPort-039-052-415	89.24	18.72
MolPort-039-052-600	86.05	20.02
MolPort-039-338-651	84.27	18.20
MolPort-039-338-719	88.40	18.58
MolPort-039-338-750	91.20	20.48
MolPort-039-339-000	92.40	15.95
MolPort-039-339-001	92.40	16.91

^(a)^ HIA%= percentage of human intestinal absorption; ^(b)^ P_Caco-2_= permeability of differentiated cells of the intestinal epithelium Caco-2 (nm·s^−1^).

**Table 5:** Predicted distribution properties of the structures.

Chemical Structure	Distribution	
	PPB(%) ^(a)^	C_Brain_/C_Blood_^(b)^
Tetrahydroxybutanefuran (pivot)	97.73	5.09
Oxacillin	61.92	0.03
Methicillin	56.05	0.11
MolPort-001-741-320	100.00	0.76
MolPort-001-742-504	100.00	2.38
MolPort-005-945-312	100.00	2.35
MolPort-005-945-435	100.00	3.20
MolPort-028-610-187	100.00	2.78
MolPort-028-610-188	98.76	3.43
MolPort-035-706-255	100.00	0.83
MolPort-035-706-257	100.00	1.28
MolPort-035-706-258	100.00	1.78
MolPort-035-706-259	100.00	1.78
MolPort-039-052-414	100.00	3.33
MolPort-039-052-415	100.00	0.19
MolPort-039-052-600	100.00	1.41
MolPort-039-338-651	100.00	1.49
MolPort-039-338-719	100.00	3.83
MolPort-039-338-750	100.00	6.62
MolPort-039-339-000	100.00	4.66
MolPort-039-339-001	100.00	5.63

^(a)^ Plasma protein binding (%); ^(b)^ blood–brain barrier permeation.

**Table 6:** Prediction of antibacterial activity via PASS Online and Antibac-Pred.

Antibacterial Activity						
Pass Online				Antibac-Pred		
Structure	Pa ^(a)^	Pi ^(b)^	Code	Name	Conf. ^(c)^	ChEMBL ID
Tetrahydroxybenzofuran	0.465	0.020	-	*S. aureus*	0.116	CHEMBL352
				RESISTANT *S. aureus*	0.062	CHEMBL352
				RESISTANT *S. aureus* subsp. *aureus* RN4220	0.948	CHEMBL2366906
Oxacillin				RESISTANT *S. aureus* subsp. *aureus* RN4220	0.948	CHEMBL2366906
	0.684	0.005	-	*S. aureus*	0.398	CHEMBL352
				*S. aureus* subsp. *aureus* RN4220	0.213	CHEMBL2366906
Methicillin	0.671	0.005	-	RESISTANT *S. aureus* subsp. *aureus* RN4220	0.878	CHEMBL2366906
				*S. aureus*	0.344	CHEMBL352
				*S. aureus* subsp. aureus RN4220	0.190	CHEMBL2366906
				RESISTANT *S. aureus*	0.032	CHEMBL352
MolPort-001-741-320	0.487	0.018	LB320	RESISTANT *S. aureus subsp. aureus* RN4220	0.059	CHEMBL2366906
				*Staphylococcus simulans*	0.362	CHEMBL612425
				*Staphylococcus sciuri*	0.353	CHEMBL613150
MolPort-035-706-255	0.344	0.045	LB255	RESISTANT *S. simulans*	0.310	CHEMBL612425
				*S. sciuri*	0.210	CHEMBL613150
				*S. simulans*	0.155	CHEMBL612425
MolPort-039-052-415	0.400	0.30	LB415	-	-	-

^(a)^ Pa = probability of being active. ^(b)^ Pi = probability of being inactive. ^(c)^ Confidence.

**Table 7:** Binding affinity and interactions of promising molecules and complexed ligand in the active site of bacterial thymidylate kinase enzyme (TMK) (PDB ID 4GSY).

Structures	ΔG ^(a)^	Hydrogen Bond (Å Distance)	Hydrophobic Interactions
Complex (0Y5)	−9.185	ARG48 (2.93) (3.02), PHE66 (5.36), ARG70 (2.89), SER97 (2.67) and GLN101 (2.79) (2.83)	PRO38, ARG48, LEU52, PHE66, ARG92 and TYR100
LB255	−7.870	GLU62 (2.15), ARG92 (3.14) and ARG105 (4.08)	PHE66 and TYR100
LB320	−8.184	ASP156 (1.61)	PHE66, ARG92 and TYR100
LB415	−8.048	LYS15 (3.37), ARG48 (5.08), ARG92 (5.13) and GLN101 (2.68) (2.71)	LEU52, PHE66, ARG92 and TYR100

^(a)^ Binding energy of the best conformation (Kcal/mol).

**Table 8:** Binding affinity and interactions of promising molecules and ligand complexed at the active site of penicillin binding protein (PBP2a-MRSA) (PDB ID 4CJN).

Structures	ΔG ^(a)^	Hydrogen Bond (Å Distance)	Hydrophobic Interactions
Complex (QZN)	−8.046	TYR105 (4.18), GLU294 (2.64) and LYS316 (2.94)	ASN146 and TYR297
LB255	−7.199	LYS273 (2.84), ASP275 (2.69) and ASP295 (1.66) (2.93)	TYR105 and TYR144
LB320	−8.019	ASN104 (2.82), TYR105 (4.14), ILE144 (2.77) and LYS273 (2.87)	ILE144 and LYS273
LB415	−7.691	ASN104 (3.37), ASN146 (2.49) (2.74), ASP295 (2.53), GLY296 (2.88) and LYS316 (2.81)	TYR105 and TYR297

^(a)^ Binding energy of the best conformation (Kcal/mol).

**Table 9:** Binding free energy for systems.

Compounds	System TMK	System PBP2a
Complex	−30.54	−32.87
LB255	−35.84	−29.15
LB320	−38.54	−36.52
LB415	−33.42	−35.76

**Table 10:** Synthetic accessibility (SA) prediction for selected compounds.

Compound	SA (%) ^(a)^	SA SCORE (%) ^(b)^
Pivot	51.31	40.98
LB255	36.58	50.17
LB320	65.08	30.88
LB415	38.88	50.06

^(a)^ AMBIT program ranges easy (score ≥ 50), median (10 < score ≤ 49), and difficult (score ≤ 10); ^(b)^ SwissADME—SA scores range from 10 (very easy) to 100 (very difficult).

**Table 11:** Prediction of lipophilicity (Log*P*_o/w_) through the SwissADME web server.

Chemical Structure	iLOG*P*	XLOG*P*3	WLOG*P*	MLOG*P*	SILICOS-IT	Mean
Methicillin	2.24	1.22	0.57	1.01	0.78	1.16
Oxacillin	2.23	2.38	1.51	1.56	1.59	1.85
LB255	3.31	1.48	1.68	0.78	1.69	1.79
LB320	2.98	4.61	3.23	2.13	3.38	3.27
LB415	1.14	2.56	2.62	1.14	4.26	2.34

**Table 12:** Prediction of water solubility (Log*S*) using the SwissADME web server.

Chemical Structure	ESOL	Ali	SILICOS-IT	Mean
Methicillin	−2.74	−3.56	−2.75	−3.01
Oxacillin	−3.79	−4.92	−4.23	−4.31
LB255	−2.83	−3.11	−1.57	−2.50
LB320	−5.26	−6.75	−2.32	−4.77
LB415	−3.85	−4.92	−3.14	−3.97

**Table 13:** Selected structures and their respective biological activity values (MIC) for ATCC 25923 *S. aureus* (MSSA).

Nº	Code SMILES	MIC ^(a)^
01	C[C@H](CC)C(=O)c1c2oc3c(c2c(O)c(CCC)c1O)c(O)c(CCC)c(O)c3C(=O)[C@H](C)CC	0.24
02	Oc1c(c(O)c(c(O)c1CCC)c1c(O)c(CCC)c(O)c(C(=O)C(C)CC)c1O)C(=O)C(C)CC	2.00
03	C[C@@H](CC)C(=O)c1c2oc3c(c2c(O)c(CCC(C)C)c1O)c(O)c(CCC(C)C)c(O)c3C(=O)[C@H](C)CC	2.16
04	C[C@H](CC)C(=O)c1c2oc3c(c2c(O)cc1O)c(O)cc(O)c3C(=O)[C@@H](C)CC	3.20
05	Oc1c(c(O)c(c(O)c1CCC(C)C)c1c(O)c(CCC(C)C)c(O)c(C(=O)C(C)CC)c1O)C(=O)C(C)CC	4.64
06	Oc1c(c(O)cc(O)c1CCC(C)C)C(=O)C(C)CC	8.96
07	O=C(c1c(O)cc(O)cc1O)C(C)CC	13.44
08	Oc1c(c(O)cc(O)c1CCC)C(=O)C(C)CC	16.13
09	Oc1cc2oc3cc(O)cc(O)c3c2c(O)c1	>30

^(a)^ MIC = µg/mL.

**Table 14:** Coordinates of active sites of molecular targets.

Receptor	Ligand/ID	Grid Center Coordinates	Grid Box Dimensions
Penicillin-binding protein (PBP2a-MRSA) PDB ID: 4CJN	(E)-3-(2-(4-Cyanostyryl)-4-oxoquinazolin-3(4H)-yl)benzoic acid/QZN	X= 8.992933 Y= −1.203300 Z= −69.561400	20x 20y 20z
Thymidylate kinase enzyme (TMK) PDB ID: 4GSY	4-{[(3S)-3-(5-Methyl-2,4-dio-xo-3,4-dihydropyr-midi-1(2H)yl)piperidin-1-yl]methyl}-2-[3(triflu-oromethyl)phenoxy]benzoic acid/0Y5	X= 8.577139 Y= 0.252556 Z= 27.171667	20x 20y 20z

## Data Availability

Data is contained within the article and supplementary material.

## Supplementary Materials

The following supporting information can be downloaded at: https://www.mdpi.com/article/10.3390/ph16101430/s1, Table S1. Spatial Coordinates of the Pharmacophoric Model.
