# Supplementary material for: Hierarchical Virtual Screening of Potential New Antibiotics from Polyoxygenated Dibenzofurans against Staphylococcus aureus Strains
Source: Pharmaceuticals (Basel). 2023 Oct 9;16(10):1430. doi: 10.3390/ph16101430 (PMC10610096; doi:10.3390/ph16101430)
Supplement: Supplementary file 1 [file pharmaceuticals-16-01430-s001.zip › pharmaceuticals-2570114-supplementary.pdf]

pharmaceuticals

Hierarchical Virtual Screening of potential new antibiotics from polyoxygenated dibenzofurans against *Staphylococcus aureus* strains

Lana P. S. Oliveira [1,2], Lúcio R. Lima [2,3,4], Luciane B. Silva [2,4], Jorddy N. Cruz [2], Ryan S. Ramos [1,2], Luciana S. Lima [5], Francy M. N. Cardoso [1,2,5], Aderaldo V. Silva [1,2], Dália P. Rodrigues [6], Gabriela S. Rodrigues [7] Aldo A. Proietti-Junior [1,5], Gabriela B. dos Santos[7], Joaquín M. Campos [8] and Cleydson B. R. Santos [1,2,3]*

[1] Graduate Program in Biotechnology and Biodiversity-Network BIONORTE, Federal University of Amapá, Macapá, 68903-419, AP, Brazil.
[2] Laboratory of Modeling and Computational Chemistry, Department of Biological and Health Sciences, Federal University of Amapá, Macapá 68902-280, AP, Brazil.
[3] Graduate Program in Network in Pharmaceutical Innovation, Federal University of Amapá, Macapá, 68902280, AP, Brazil.
[4] Graduate Program in Medicinal Chemistry and Molecular Modeling, Health Science Institute, Federal Univesity of Pará, Belém 66075-110, Brazil.
[5] Special Laboratory of Applied Microbiology, Department of Biological and Health Sciences, Federal University of Amapá, Macapá 68902-280, AP, Brazil.
[6] Laboratory of Bacterial Enteric Pathogens, Oswaldo Cruz Institute, FIOCRUZ, 21045-900, Rio de Janeiro, Brazil.
[7] Graduate Program in Health Sciences, Institute of Collective Health, Federal University of Western Pará, Santarém, 68270-000, PA, Brazil.
[8] Department of Pharmaceutical and Organic Chemistry, Faculty of Pharmacy; Institute of Biosanitary Research ibs. GRANADA. University of Granada, 18071, Granada, Spain.

***** Correspondence: Cleydson B. R. Santos (breno@unifap.br)

Supplementary Material

Table S1. Spatial Coordinates of the Pharmacophoric Model.

Table S1. Spatial Coordinates of the Pharmacophoric Model.

Pharmacophoric Characteristics		Coordinated			
		X	Y	Z	Radius
Aromatic	ARO	9.426	-7.548	-0.150	1.10
Hydrogen Acceptor	ACC1	11.901	-8.764	-0.087	0.50
Hydrogen Acceptor	ACC2	11.873	-4.029	0.064	0.50
Hydrogen Acceptor	ACC3	6.956	-8.825	-0.394	0.50
Hydrogen Acceptor	ACC4	9.563	-4.761	-0.045	0.50
Hydrogen Donor	DON1	6.956	-8.825	-0.394	0.50
Hydrogen Donor	DON2	9.563	-4.761	-0.045	0.50
